# Repurposing potential of posaconazole and grazoprevir as inhibitors of SARS-CoV-2 helicase

**DOI:** 10.1038/s41598-021-89724-0

**Published:** 2021-05-13

**Authors:** Syed Hani Abidi, Nahlah Makki Almansour, Daulet Amerzhanov, Khaled S. Allemailem, Wardah Rafaqat, Mahmoud A. A. Ibrahim, Philip la Fleur, Martin Lukac, Syed Ali

**Affiliations:** 1 Department of Biological and Biomedical Sciences, Aga Khan University, Karachi, Pakistan; 2 Department of Biology, College of Science, University of Hafr Al Batin, Hafr Al Batin, Saudi Arabia; 3 Nazarbayev University School of Medicine, Nazarbayev University, Astana, Kazakhstan; 4 Department of Medical Laboratories, College of Applied Medical Sciences, Qassim University, Buraydah, Saudi Arabia; 5 Medical College, Aga Khan University, Karachi, Pakistan; 6 Computational Chemistry Laboratory, Chemistry Department, Faculty of Science, Minia University, Minia, 61519 Egypt; 7 Department of Computer Science, School of Engineering and Digital Sciences, Nazarbayev University, Astana, Kazakhstan

**Keywords:** Drug screening, Medicinal chemistry, Target identification, Target validation, Nucleic-acid therapeutics, SARS-CoV-2

## Abstract

As the Severe Acute Respiratory Syndrome Coronavirus-2 (SARS-CoV-2) pandemic engulfs millions worldwide, the quest for vaccines or drugs against the virus continues. The helicase protein of SARS-CoV-2 represents an attractive target for drug discovery since inhibition of helicase activity can suppress viral replication. Using in silico approaches, we have identified drugs that interact with SARS-CoV-2 helicase based on the presence of amino acid arrangements matching binding sites of drugs in previously annotated protein structures. The drugs exhibiting an RMSD of ≤ 3.0 Å were further analyzed using molecular docking, molecular dynamics (MD) simulation, and post-MD analyses. Using these approaches, we found 12 drugs that showed strong interactions with SARS-CoV-2 helicase amino acids. The analyses were performed using the recently available SARS-CoV-2 helicase structure (PDB ID: 5RL6). Based on the MM-GBSA approach, out of the 12 drugs, two drugs, namely posaconazole and grazoprevir, showed the most favorable binding energy, − 54.8 and − 49.1 kcal/mol, respectively. Furthermore, of the amino acids found conserved among all human coronaviruses, 10/11 and 10/12 were targeted by, respectively, grazoprevir and posaconazole. These residues are part of the crucial DEAD-like helicase C and DEXXQc_Upf1-like/ DEAD-like helicase domains. Strong interactions of posaconazole and grazoprevir with conserved amino acids indicate that the drugs can be potent against SARS-CoV-2. Since the amino acids are conserved among the human coronaviruses, the virus is unlikely to develop resistance mutations against these drugs. Since these drugs are already in use, they may be immediately repurposed for SARS-CoV-2 therapy.

## Introduction

Severe Acute Respiratory Syndrome Coronavirus-2 (SARS-CoV-2), responsible for the ongoing global pandemic, causes a respiratory infection found potentially fatal among elderly and immune-compromised patients^1,2^. SARS-CoV-2 is a double-stranded, positive-sense RNA virus that belongs to the Coronaviridae family. The Coronavirus (CoV) genome frequently undergoes recombination and can produce novel strains with variations in virulence^3^. There are seven strains of human coronaviruses (HCoV), namely, HCoV-229E, HCoV-NL63, HCoV-OC43, HCoV-HKU1, Middle East respiratory syndrome (MERS)-CoV, severe acute respiratory syndrome (SARS-CoV), and the 2019 novel coronavirus (nCoV) or SARS-CoV-2^4–6^. The SARS-CoV and the MERS-CoV have been responsible for large-scale epidemics in 2003 and 2012, respectively^3^. As the SARS-CoV-2 pandemic engulfs millions around the world, there is a struggle to find an effective vaccine or drug against the virus. While several drugs, including oseltamivir, lopinavir, ritonavir, arbidol, and chloroquine, have been tried with limited success, the search for effective therapy is still underway^7–11^.

Due to the pivotal role that helicases play in the viral life cycle, they represent an attractive target for antiviral therapy. To separate nucleic acid strands, energy derived from ATP hydrolysis is utilized by helicases, nucleic acid unwinding motor proteins. This process is crucial for genome replication^12^, transcription of viral mRNAs, translation, disruption of RNA–protein complexes, and packaging of nucleic acids into virions^12^. Depending on whether they can bind single-stranded (ss) nucleic acid, unwind double-stranded (ds) RNA or dsDNA or both, the polarity of the unwinding (5′ to 3′ or 3′ to 5′), and whether specific signature motifs are present in their primary sequence, helicases are classified into six superfamilies (SF1–SF6)^13^. Helicases belonging to SF1 and SF2 generally act as monomers or dimers on DNA or RNA substrates, whereas most of the SF3–SF6 helicases form ring-shaped hexameric structures that encircle the nucleic acid and have roles mainly in DNA replication^14,15^. SARS-CoV-2 helicase enzyme is a member of the SF1 that prefers ATP, dATP, and dCTP as substrates, while hydrolyzing other NTPs as well^12,16^.

Several viral helicases have been used as targets in animal models of herpes simplex (HSV) and hepatitis C (HCV) viruses^17,18^. The importance of helicase validity as antiviral drug targets was recently corroborated when compounds that inhibit an HSV helicase were shown to block viral replication and disease progression in animal models^19^. Similarly, much effort has been directed towards developing small-molecule inhibitors and chemicals as drug candidates to inhibit the function of SARS-CoV-1 helicase nsP13 (SCV nsP13)^17,20^. Unlike the Spike protein that is the key target for antibody-based therapeutics, the nsp13 helicase protein of SARS-CoV-2, perhaps owing to its pivotal role in the virus life cycle, is quite conserved among the human coronavirus family^21^. The conservation and functional importance of helicase makes it an ideal target for antiviral drugs.

Here, using in silico approaches, including homology modeling, molecular docking, and molecular dynamic simulations, we found a panel of 12 drugs that show strong interactions/affinity with SARS-CoV-2 helicase amino acids. The amino acids targeted by the drugs are highly conserved and appear to be crucial for helicase function, indicating that the drugs will be potent against SARS-CoV-2 and that the virus is unlikely to develop resistance mutations against these drugs. Since these drugs are currently used for antiviral and chemotherapeutic purposes, they can be repurposed to treat SARS-CoV-2 without an extensive drug safety profiling process. This will especially benefit regions without high-level biosafety facilities for testing viral drugs, will and provide a timely solution for SARS-CoV-2 therapy^22^.

## Computational methodology

### Sequence retrieval, analysis of domain architecture, and conservation

SARS-CoV-2 helicase amino acid sequence was retrieved in FASTA format from the National Center for Biotechnology (NCBI) Genbank (NCBI genome ID: MN908947). Conserved domains in the retrieved structure were mapped using the NCBI Conserved Domain Search (CDD) tool tool v3.19^23^. Conserved Domain Architecture Retrieval Tool (CDART) and Subfamily Protein Architecture Labeling Engine (SPARCLE) tools were used to identify sequences sharing of domain architecture with our query (SARS-CoV-2 helicase) sequence, and the search was refined to identify only human virus sequences^24^.

### Retrieval of SARS-CoV-2 helicase structure

For our analysis, experimental 3D structure of SARS-CoV-2 helicase (PDB ID: 5RL6) was retrieved from PDB. The structure was visually inspected in Discovery Studio Visualizer version 4.0 (DSV4.0; Dassault Systèmes BIOVIA, Discovery Studio Visualizer). Subsequently, the structure was verified using the Verify 3D tool, while the energy minimization and validation were performed using the GROMACS, ERAAT, Verify3D, and Ramachandran plot analysis implemented in DSV4.0^25–27^.

### Prediction of drug that can interact with SARS-CoV-2 helicase protein and retrieval of drug structures

Drug ReposER tool, a web server that uses a modified version of the SPRITE search engine to identify similar amino acid arrangements to known drug binding interfaces for potential drug repositioning, was used to predict/identify drugs that could interact with the SARS-CoV-2 helicase based on the presence of amino acid arrangements matching binding sites of drugs in previously annotated protein structures^28^. The tool predicts the binding of drugs with query protein based on RMSD. We used RMSD of 3.0 Å or less as the threshold, and structures of drugs exhibiting RMSD 3.0 Å and under were retrieved from PubChem Database in 3D SDF format. The 3D geometrical structures of drugs were then minimized by the Merck Molecular Force Field 94 (MMFF94S) force field using SZYBKI software^29,30^. Before docking analysis, SDF structures were converted to PDBQT format using the OpenBabel tool, and polar hydrogens were added to the drug structures during conversion^31^.

### Drug-protein docking

The protonation state of SARS-CoV-2 helicase was first investigated using the H^++^ server^32^. In H^++^ calculations, the following physical conditions were employed: pH = 6.5, internal dielectric = 10, external dielectric = 80 and salinity = 0.15. The SARS-CoV-2 helicase was then prepared based on the AutoDock protocol^33^. The preparation involved merging of nonpolar hydrogens, addition of polar hydrogens, and generation of PDBQT files using AutoDock Tools^34^. Subsequently, molecular docking calculations were performed to predict and analyze the drug-helicase interactions using AutoDock Vina software^35^. The Vina parameters were kept to the default, except the exhaustiveness parameter was set to 200. Blind docking was employed in which the binding site was realized by a docking box around the whole protein. The grid spacing value was set to 1.0 Å. Visualization of docking poses and analysis of drug-protein interactions was performed using Discovery Studio Visualizer version 4.0 (DSV4.0; Dassault Systèmes BIOVIA, Discovery Studio Visualizer).

### MD simulations and MM-GBSA energy calculations

Molecular dynamics (MD) simulations for the repurposed drugs complexed with SARS-CoV-2 helicase were performed using AMBER16 software^36^. Two AMBER force fields were used to describe the drug and helicase —namely, general AMBER force field (GAFF)^37^ and AMBER force field 14SB^38^, respectively. The atomic partial charges of the repurposed drugs were assigned using the restrained electrostatic potential (RESP) approach^39^ at the HF/6-31G* level with the assistance of Gaussian09 software^40^. The docked drug-helicase complexes were water solvated with 15 Å distances between the box edge and atoms of the solute. The solvated systems were minimized by 5000 steps and afterward gently heated from 0 to 300 K over 50 ps. Using periodic boundary conditions and NPT ensemble, the systems were equilibrated for 1 ns, and production stages of 100 ns were executed. Particle Mesh Ewald (PME) method^41^ with a direct space cut-off of 12 Å was employed to treat the long-range electrostatic interactions. Langevin dynamics with a gamma_ln parameter of 1.0 was adopted to retain the temperature constant at 298 K. Berendsen barostat with a relaxation time of 2 ps was employed to control the pressure of the system^42^. All bonds involving hydrogen atoms were constrained using the SHAKE option, and the time step was set to 2 fs. Over the production stage, uncorrelated snapshots were collected over every 20 ps for binding energy calculations. The binding energies were calculated using the molecular mechanical-generalized Born surface area (MM-GBSA) approach^43^ with a modified GB model (igb = 2) implemented in AMBER16 software. The binding energy (Δ*G*_*binding*_) was evaluated as follows:

$$\Delta G_{binding} = G_{drug - helicase} {-} \, \left( {G_{drug} + \, G_{helicase} } \right)$$

where the energy term (G) is estimated as:

$$G \, = \, E_{vdw} + \, E_{ele} + \, G_{GB} + \, G_{SA}$$

*E*_*vdw*_ and *E*_*ele*_ are van der Waals and electrostatic energies, respectively. *G*_*GB*_ is the electrostatic solvation free energy calculated from the generalized Born equation and *G*_*SA*_ is the nonpolar contribution to the solvation free energy from the solvent-accessible surface area (SASA). All molecular dynamics simulations were executed with pmemd.cuda implemented in AMBER16. All molecular docking and molecular dynamics calculations were performed on the CompChem GPU/CPU cluster (hpc.compchem.net).

## Results

### Analysis of domain architecture and conservation of domains

**Figure 1:**
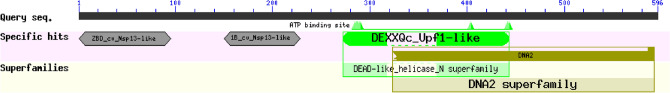
Protein and domain classification. Conserved domains in the SARS-CoV-2 structure were mapped using the NCBI Conserved Domain Search (CDD) tool v3.19. The SARS-CoV-2 helicase was found to be a DNA2 superfamily helicase with two significant domains: DEAD-like helicase C (spanning amino acids 323–592) and DEXXQc_Upf1-like (spanning amino acids 272–443), containing Walker A motif (GTGKSH) at N-terminus that is involved in ATP binding. Two additional functional domains ZBD_cv_Nsp13-like (spanning amino acids 1–95) and 1B_cv_Nsp13-like (spanning amino acids 150–228) were also found in the sequence. (*note: the figure is an original image generated by CDD v3.19 tool*).

The conserved domains in the SARS-CoV-2 helicase sequence were mapped using the NCBI CDD tool, and the CDART and SPARCEL tool was used to identify sequences sharing domain architecture with our query (SARS-CoV-2 helicase) sequence. The SARS-CoV-2 helicase was found to be a DNA2 superfamily helicase with two significant domains: DEAD-like helicase C (cd17934) and DEXXQc_Upf1-like (accession number COG1112), containing Walker A motif at N-terminus that is involved in ATP binding (Fig. 1). Two additional functional domains, ZBD_cv_Nsp13-like (spanning amino acids 1–95) and 1B_cv_Nsp13-like (spanning amino acids 150–228), were also found in the query sequence (Fig. 1). Analysis of the conserved domain architecture (architecture ID: 13027813) suggested that the DEXXQc_Upf1-like and DEAD-like_helicase_C domains were conserved in helicases from 70 different organisms, including humans, fungi, bacteria, and viruses. Analysis of only viral sequences suggested that DEXXQc_Upf1-like and DEAD-like_helicase_C domains were conserved features of coronavirus helicase, where helicase from 28 different coronaviruses, including SARS and MERS, were found to possess the DEXXQc_Upf1-like and DEAD-like_helicase_C domain (Table 1). The sequences from human coronaviruses were used for further analysis.

**Table 1:** Domain architecture conservation in viruses.

No	Identifier	Description	Organism
1	ACU31046	Helicase, partial	Bat SARS-CoV Rs806/2006
2	YP_008439223	nsp13	Bat coronavirus CDPHE15/USA/2006
3	YP_459942	nsp13	Human coronavirus HKU1
4	NP_742139	Coronavirus nsp10 (MB, NTPase/HEL)	Bovine coronavirus
5	ABB77060	Helicase, partial	Pipistrellus bat coronavirus HKU5
6	ABB77058	Helicase, partial	Pipistrellus bat coronavirus HKU5
7	ABB77061	Helicase, partial	Bat coronavirus HKU6
8	ABB77050	Helicase, partial	Rhinolophus bat coronavirus HKU2
9	ABB77053	Helicase, partial	Tylonycteris bat coronavirus HKU4
10	ABB77054	Helicase, partial	Tylonycteris bat coronavirus HKU4
11	ABB77057	Helicase, partial	Pipistrellus bat coronavirus HKU5
12	ABB77056	Helicase, partial	Pipistrellus bat coronavirus HKU5
13	ABB77051	Helicase, partial	Rhinolophus bat coronavirus HKU2
14	ABB77052	Helicase, partial	Tylonycteris bat coronavirus HKU4
15	ABB77055	Helicase, partial	Tylonycteris bat coronavirus HKU4
16	NP_839966	Putative coronavirus nsp10 (MB, NTPase/HEL)	Porcine epidemic diarrhea virus
17	YP_209240	nsp13; zinc-binding domain and helicase	Murine hepatitis virus strain JHM
18	ABD15361	HELICASE, partial	Miniopterus bat coronavirus HKU8
19	YP_009555254	nsp10	Human coronavirus OC43
20	ABO88148	Helicase, partial	Bat coronavirus Anhui/911/2005
21	ABG11967	Helicase, partial	Bat coronavirus (BtCoV/A434/2005)
22	ABG11968	HELICASE, partial	Bat coronavirus A515/2005
23	YP_009047224	nsp13 protein	Middle East respiratory syndrome-related coronavirus
24	NP_828870	nsp13-pp1ab (ZD, NTPase/HEL)	Severe acute respiratory syndrome-related coronavirus
25	ABG11969	Helicase, partial	Bat coronavirus A527/2005
26	ABG11966	Helicase, partial	Bat coronavirus (BtCoV/355A/2005)
27	5WWPA	Chain A, Crystal Structure Of Middle East Respiratory Syndrome Coronavirus Helicase (MERS-CoV Nsp13)	Human betacoronavirus 2c EMC/2012
28	5WWPB	Chain B, Crystal Structure Of Middle East Respiratory Syndrome Coronavirus Helicase (MERS-CoV Nsp13)	Human betacoronavirus 2c EMC/2012

CDART and SPARCLE tools were used to identify sequences sharing of domain architecture with our query (SARS-CoV-2 helicase) sequence. The search was refined to identify only human virus sequences, identified by NCBI identifier in column 2, which are shown in the Table. The Table also describes the nature of protein in all the shortlisted sequences and the organism to which they belong.

### Analysis of the drug-protein docking revealed strong binding affinities of drugs with the SARS-CoV-2 helicase

**Figure 2:**
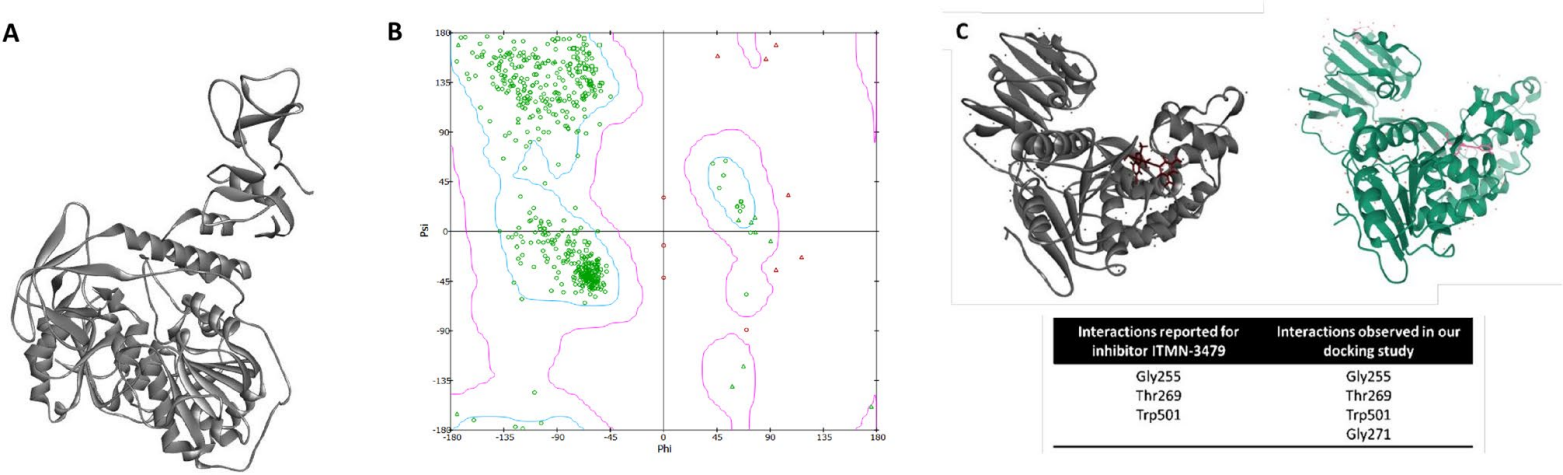
Validation of structure and docking strategy: (**A**) Structure of SARS-CoV-2 helicase used in the study, (**B**) Ramachandran plots for SARS-CoV-2 helicase structure (PDB ID: 5RL6) used in the study, and (**C**) Docking strategy was validated by re-docking a previously published inhibitor ITMN-3479 on its receptor. Poses of ligand bound to the receptor generated after docking in our study (left, ligand, and protein are shown in red and dark grey, respectively) and retrieved from PDB (right; ligand and protein are shown in pink and green, respectively) are shown, while the bottom panel shows amino acid interactions reported for each ligand and observed in our study.

Before docking, the SARS-CoV-2 structure (Fig. 2A) was validated using ERAAT, VERIFY 3D, GROMACS, and Ramachandran plot analysis. The structure passed the 3D verification (performed using Verify 3D software), with 92.83% of the residues averaged 3D-1D score ≥ 0.2. The ERAAT quality score for the structure was 90.64. The structures were also found valid on the Ramachandran plot as most (99.6%) of the amino acids were under the permissible (Fig. 2B).

Further, validation of the docking approach was confirmed by performing blind docking (assuming drug binding site to be anywhere in the protein) on a previously reported complex of HCV NS3 helicase bound to inhibitor ITMN-3479 (PDB ID: 3RVB)^44^. Our results revealed that the observed binding site/pose and drug-protein interaction were the same as reported in the crystal structure of the complex, indicating that the docking strategy was efficient and valid (Fig. 2C).

**Figure 3:**
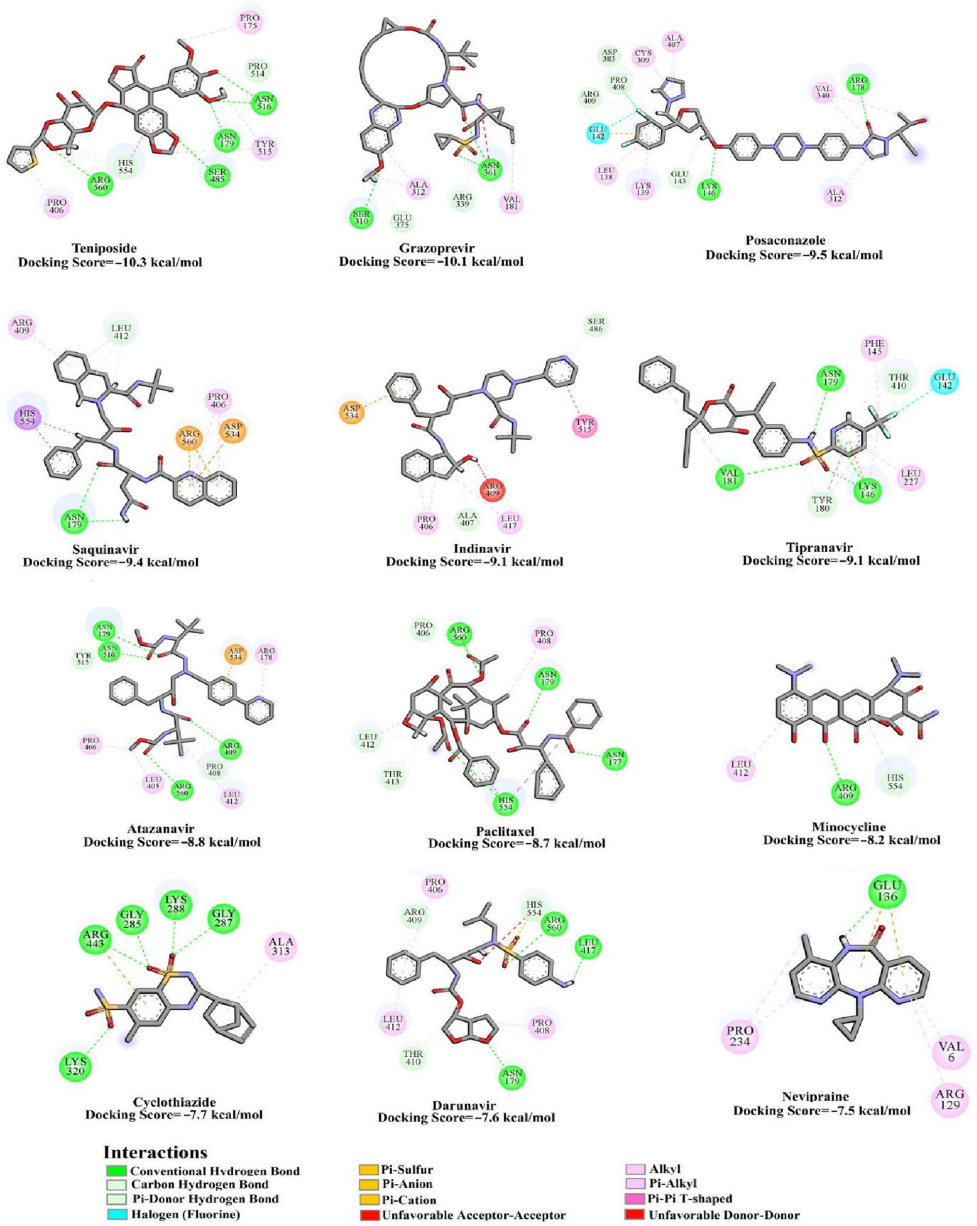
2D representations of the predicted binding modes and scores of the investigated twelve drugs inside the active site of the SARS-CoV-2 helicase.

Drug ReposER tool was used to predict/identify drugs that could interact with the SARS-CoV-2 helicase based on the presence of amino acid arrangements matching binding sites of drugs in for previously annotated protein structures. In the first step, the SARS-CoV-2 structure was loaded to the server that used PDB coordinate files to search and compare amino acid side chain arrangements that match those found in drug binding sites in previously annotated protein structures. The tool predicts the binding of drugs with query protein based on RMSD. In the next step, we set a threshold of RMSD ≤ 3.0 Å and found sites for 12 previously annotated drugs having RMSD ≤ 3.0 Å. These drugs were individually docked to the SARS-CoV-2 helicase protein analysis of the drug-protein interactions revealed that the drugs exhibited strong binding affinity with SARS-CoV-2 helicase, ranging from − 10.3 to − 7.5 kcal/mol, where teniposide, grazoprevir and posaconazole showed the lowest binding energies with a docking score of − 10.3, − 10.1 and − 9.5 kcal/mol, respectively (Fig. 3).

### MD simulations and MM-GBSA analysis

**Figure 4:**
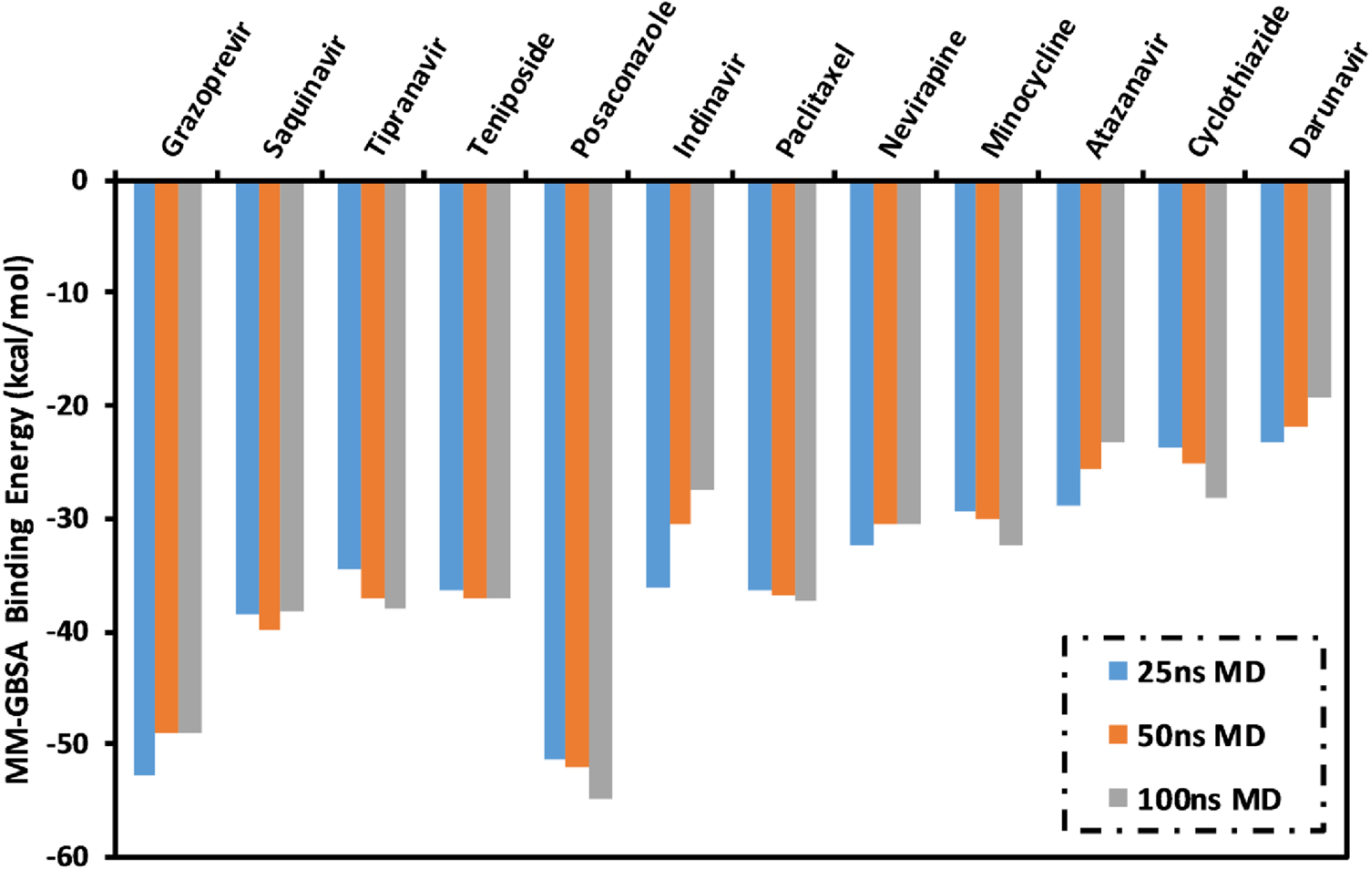
Calculated MM-GBSA binding energies for the investigated drugs as SARS-CoV-2 helicase inhibitors.

For accurate estimation of the binding affinities of the proposed drugs as SARS-CoV-2 helicase inhibitors, all docked drug-helicase complexes were solvated and subjected to molecular dynamics (MD) simulation of 100 ns. Based on the collected snapshots, binding energies were estimated using the molecular mechanics-generalized Born surface area (MM-GBSA) approach over the first 25, 50, and 100 ns MD simulations (Fig. 4). As shown in Fig. 4, among the examined drugs as potential SARS-CoV-2 helicase inhibitors, posaconazole and grazoprevir exhibited the most promising binding affinities towards SARS-CoV-2 helicase. The estimated MM-GBSA binding energies of posaconazole and grazoprevir were nearly constant over the MD course, with values of − 49.4 and − 48.1, − 51.3 and − 52.7, and − 54.8 and − 49.1 kcal/mol over 25, 50, and 100 ns MD, respectively. The surpass potentiality of grazoprevir as a SARS-CoV-2 helicase inhibitor is returned to its capability to exhibit multiple hydrogen bonds, van der Waals interactions in addition to hydrophobic and pi-based interactions with the key amino acids within the active site (Fig. 3). More precisely, grazoprevir forms three hydrogen bonds with ASN177, THR413, and ARG560 amino acid with bond lengths of 2.65, 1.91, and 2.88 Å, respectively.

To analyze the principle interactions in posaconazole- and grazoprevir-SARS-CoV-2 helicase complexes, MM-GBSA binding energy decomposition was executed over the MD course of 100 ns (Table 2). Interestingly, Vander Waals energy (*E*_vdw)_ was found to be the predominant component in the interactions of posaconazole- and grazoprevir with SARS-CoV-2 helicase complexes, with binding energies of − 77.9 and − 68.7 kcal/mol, respectively (Table 2). Additionally, for the two drug-protein complexes, the electrostatic energies (*E*_*ele*_) _of_− 24.6 and − 28.4 kcal/mol, respectively, were also favorable (Table 2).

**Table 2:** MM-GBSA binding energies decomposition for the top two investigated drugs in complex with SARS-CoV-2 helicase through the MD course of 100 ns.

Drug	Estimated MM-GBSA binding energy (kcal/mol)						
	∆*E*_VDW_	∆*E*_ele_	∆*E*_GB_	∆*E*_SUR_	∆*G*_gas_	∆*G*_solv_	∆*G*_binding_
Posaconazole	− 77.9	− 24.6	56.8	− 9.1	− 99.8	47.7	− 54.8
Grazoprevir	− 68.7	− 28.4	56.7	− 8.7	− 96.9	48.0	− 49.1

### Post-dynamics analyses

To evaluate the stability of the interaction of the posaconazole and grazoprevir inside the active site of SARS-CoV-2 helicase, structural and energetic analyses were carried out over the 100 ns MD simulations. Analyses involve binding energy per frame, hydrogen bond lengths, and root-mean-square deviation (RMSD).

#### Binding energy per frame

**Figure 5:**
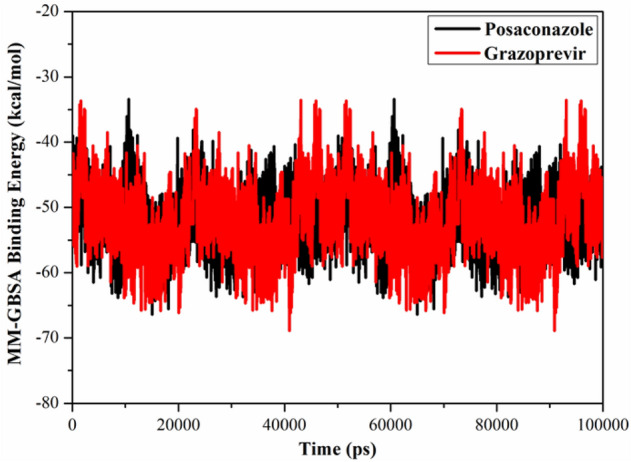
Evaluated MM-GBSA binding energy per frame for posaconazole (in black) and grazoprevir (in red) towards SARS-CoV-2 helicase throughout 100 ns MD simulation.

The stability of posaconazole and grazoprevir inside the SARS-CoV-2 helicase active site was scrutinized via inspecting the correlation between the binding energy per frame and time (Fig. 5). The most exciting aspect of the data illustrated in Fig. 5 was the overall stability of posaconazole and grazoprevir towards SARS-CoV-2 helicase through the MD course of 100 ns with average values − 54.8, − 49.1 kcal/mol, respectively.

#### Hydrogen bond length

Hydrogen bond analysis was executed on the production MD trajectory, and the results are presented in Table 3. What stands out in Table 3 is the high stability of two identified drugs towards SARS-CoV-2 helicase. Posaconazole and grazoprevir form an essential hydrogen bond with ASP315 and LEU141 with an average bond length of 2.7 and 2.8 Å, respectively (Table 3). The posaconazole and grazoprevir showed a persistent 95.6 and 93.9% of the production MD trajectory snapshots, respectively (Table 3). Overall, these post-dynamics outcomes illustrated proof for the stability of posaconazole and grazoprevir in complex with SARS-CoV-2 helicase.

**Table 3:** Hydrogen bonds exhibited between the key residues and the most promising drugs against SARS-CoV-2 helicase.

Drug	Acceptor	Donor	Distance (Å)^a^	Angle (°)^a^	Occupied (%)^b^
Posaconazole	ASP315@OD1	Posaconazole597@O2-H41	2.7	164	95.6
Grazoprevir	LEU412@O	Grazoprevir597@N4-H48	2.8	161	93.9

^a^The hydrogen bonds are inspected by the acceptor–donor atom distance of < 3.5 Å and acceptor-H-donor angle of > 120°.

^b^Occupancy is employed to estimate the stability and strength of the hydrogen bond.

#### Root-mean-square deviation

**Figure 6:**
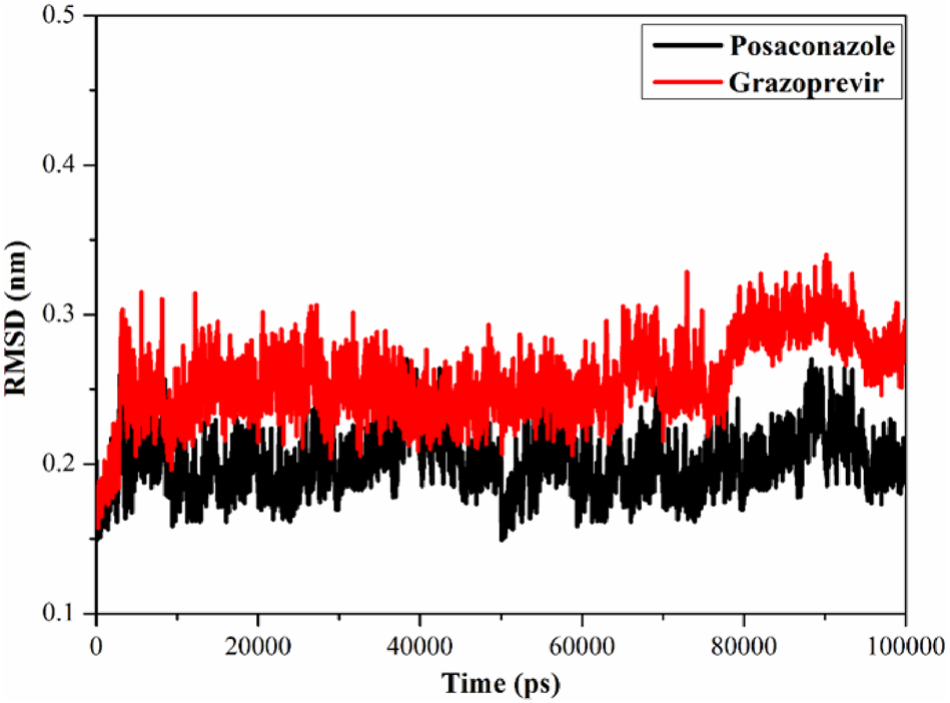
Root mean square deviation (RMSD) of the backbone atoms from the initial structure for posaconazole (in black) and grazoprevir (in red) with the SARS-CoV-2 helicase over 100 ns MD simulations.

The structural changes of posaconazole and grazoprevir in complex with SARS-CoV-2 helicase were estimated using root-mean-square deviation (RMSD). The conformational change of backbone atoms was evaluated throughout the 100 ns MD simulations and compared to the initial conformation (Fig. 6). As shown in Fig. 6, the overall stability of posaconazole and grazoprevir was observed with an average RMSD value of 0.20 and 0.26 nm, respectively. Eventually, the presented results proved that the two drugs are tightly bonded with, and do not influence the overall topology of, SARS-CoV-2 helicase.

### Drug-protein docking revealed that Posaconazole and Grazoprevir target conserved residues in functional domains of the SARS-CoV-2 helicase

**Figure 7:**
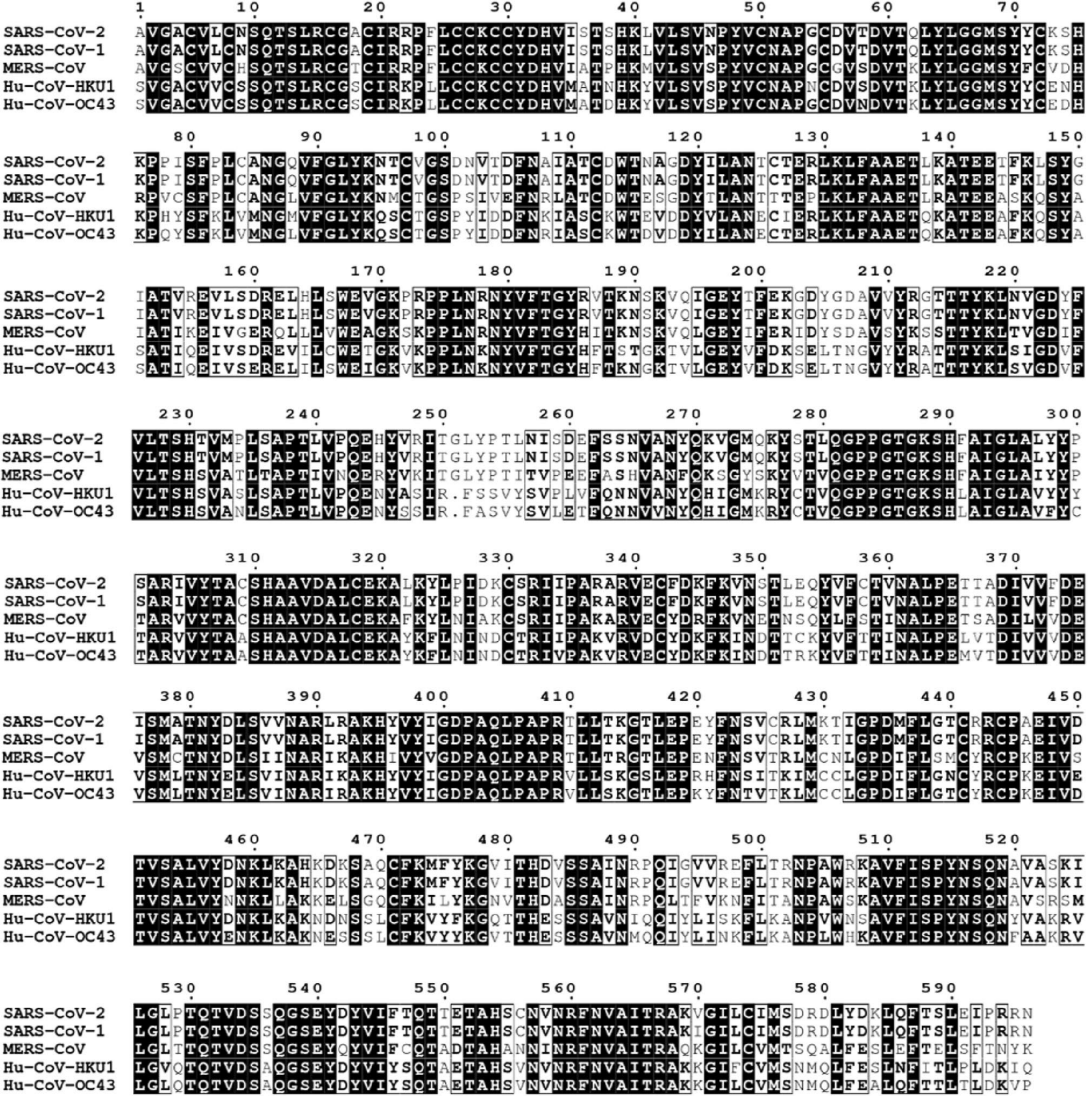
Sequence alignment of known human coronaviruses sharing helicase domain architecture: Multiple sequence alignment (ranging from amino acid 3–596, numbered according to their position in the helicase protein) was performed employing ‘Clustal W’. Conserved residues/sites are highlighted in black color, residues conserved in two or more sequences are shown in black font, while differences are shown in grey font. Publication quality alignment was prepared using the ENDscript server^45^.

Interestingly, most of the amino acids that formed interactions with posaconazole and grazoprevir are crucial for helicase activity (Fig. 1) and were found to be conserved in two or more known human coronavirus helicases (Fig. 7). Among these, 10 out of 11 residues targeted by grazoprevir were conserved among all human coronaviruses, while the remaining 1 residue (THR413) was only conserved in SARS-CoV-2, SARS-CoV-1, and MERS (Fig. 7). Similarly, 10 out 12 residues targeted by posaconazole were conserved among all human coronaviruses, while the remaining two residues (THR416 and ARG178) were only conserved in SARS-CoV-2, SARS-CoV-1, and MERS (Fig. 7). These residues targeted by both the drugs are part of DEAD-like helicase C and DEXXQc_Upf1-like/DEAD-like helicase domains (Fig. 1).

## Discussion

Here, using in silico analyses, we identify drugs that may interact and inhibit the SARS-CoV-2 helicase, nsp13. The SARS-CoV-2 structure was loaded to the server that used PDB coordinate files to search and compare amino acid side chain arrangements that match those found in the drug binding sites in previously annotated protein structures. Drugs thus identified were then shortlisted for docking studies using a threshold of RMSD ≤ 3.0 Å^46^. This approach gave us 12 drugs, namely posaconazole, grazoprevir, tipranavir, paclitaxel, saquinavir, teniposide, indinavir, atazanavir, nevirapine, minocycline, cyclothiazide, and darunavir, exhibiting strong binding with SARS-CoV-2 helicase protein. Out of these 12, posaconazole, and grazoprevir were found to be the most potent (Figs. 3 and 4).

Most of the amino acids targeted by grazoprevir and Posaconazole ((10/11 and 10/12, respectively) were conserved among all human coronaviruses. Additionally, these residues are part of the crucial DEAD-like helicase C and DEXXQc_Upf1-like/ DEAD-like domains of helicase (Table 1), indicating their key roles in the helicase activity. Upf1 domain of the helicase is crucial for nonsense-mediated mRNA decay. It retains a tight grip on nucleic acids during helicase action^47^. On the N-terminus, Upf1 has Walker A motif, which functions as a phosphate-binding P-loop used by the helicase to bind NTP using another motif, i.e. Walker B, which acts as an Mg^2+^ co-factor-binding loop^48^. The A and B motifs of Walker-type NTP-binding pattern are perhaps the only sequence elements shared by all known groups of helicases^49^. The DEAD helicases have a diverse role in all phases of RNA transcription, including mRNA splicing, export, translation, stability, etc.^50^. Although RNA helicases are large in number, yet each RNA helicase seems to have its significance in RNA processing. For example, a study performed in yeast showed that functional loss of one DEAD-box helicase could not be supplemented by another related helicase^51^. In addition to RNA transcription, DEAD-box helicases also aid in ribosome biosynthesis by mediating interactions between small nucleolar and ribosomal RNA^52^. In the SARS virus, 1B regulatory domain of Nsp13 helicase is involved in nucleic acid substrate binding.

Both grazoprevir and Posaconazole exhibited strong binding energies with the helicase protein, i.e. − 54.8 and − 49.1 kcal/mol, respectively, based on the MM-GBSA approach over the first 100 ns MD simulations. In the MM-GBSA analysis, Vander Waals forces were found to be the principal force of interaction between the drugs and protein, and the interactions were electrostatically favorable. Considering the essential nature of motifs DEXXQc_Upf1-like and DEAD-like helicase C in helicase function, it may be speculated that the drugs strongly interacting with these motifs will potently inhibit the helicase activity and will, therefore, be highly effective as antivirals.

Both the shortlisted drugs have well-established safety profiles. Posaconazole is a potent triazole antifungal drug used to treat invasive fungal infections in severely immunocompromised patients. In the clinical trial, higher doses (up to 1600 mg/day) had no adverse effects as compared to lower doses^53^. Similarly, grazoprevir is a potent antiviral drug used against HCV and inhibits HCV NS3/4A, a serine protease enzyme^53^. The adverse effects of this drug, at all intensities, are limited to fatigue, headache, and nausea^53^. Based on the well-characterized safety profiles^53^, the two drugs can be evaluated for immediate clinical use.

In conclusion, grazoprevir and posaconazole drugs show considerable potential for repurposing as antivirals against SARS-CoV-2. In the motifs, DEXXQc_Upf1-like and DEAD-like helicase C, conservation of the interacting amino acid residues throughout human coronaviruses indicates that the drugs will effectively inhibit SARS-CoV-2 helicase and that the virus is unlikely to develop resistance to these antivirals. Given the emergent need for treatment against SARS-CoV-2, we propose immediate trials to evaluate the repurposing of these two drugs as antivirals.
